# *N*-[(4-Carbamoylphen­yl)carbamothio­yl]-2,3,4,5-tetra­fluoro­benzamide

**DOI:** 10.1107/S1600536811005915

**Published:** 2011-02-23

**Authors:** Li-Dan Zhang, Chao Gao, Xue-Jiao Song, Luo-Ting Yu

**Affiliations:** a Department of Pharmaceutical and Bioengineering, School of Chemical Engineering, Sichuan University, Chengdu 610065, People’s Republic of China; b State Key Laboratory of Biotherapy and Cancer Center, West China Hospital, West China Medical School, Sichuan University, Chengdu 610041, People’s Republic of China; c West China School of Pharmacy, Sichuan University, Chengdu 610041, People’s Republic of China

## Abstract

In the title compound, C_15_H_9_F_4_N_3_O_2_S, the *N*,*N*′-disubstituted thio­urea fragment adopts a *cis*,*trans* geometry, stabilized by an intra­molecular N—H⋯O hydrogen bond to the carbonyl O atom of the tetra­fluoro­benzoyl group. The central thio­urea group makes dihedral angles of 47.79 (7) and 35.54 (8)° with the two aromatic rings. In the crystal, mol­ecules are linked *via* N—H⋯O and N—H⋯S hydrogen bonds into two-dimensional polymeric structures parallel to (100). In turn, π–π stacking inter­actions between tetra­fluoro­benzene and benzene units [centroid–centroid distance = 3.996 (10) Å; dihedral angle = 13.60 (8)°] organize these two-dimensional assemblies into a three-dimensional framework.

## Related literature

For the biological activity of thio­urea derivatives, see: Zeng *et al.* (2003 ▶); Saeed *et al.* (2010 ▶). For the synthesis of thio­urea derivatives, see: Nosova *et al.* (2007 ▶). For related structures, see: Saeed *et al.* (2008 ▶, 2009 ▶).
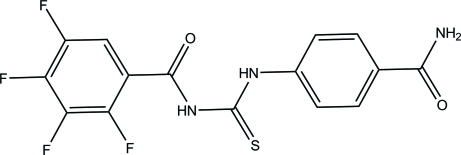


## Experimental

### 

#### Crystal data

C_15_H_9_F_4_N_3_O_2_S
*M* *_r_* = 371.31
Monoclinic, 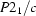

*a* = 7.4246 (3) Å
*b* = 20.3368 (7) Å
*c* = 9.8954 (4) Å
β = 95.554 (3)°
*V* = 1487.12 (9) Å^3^
*Z* = 4
Mo *K*α radiation
μ = 0.28 mm^−1^
*T* = 294 K
0.38 × 0.30 × 0.26 mm

#### Data collection

Oxford Diffraction Xcalibur E CCD diffractometer
Absorption correction: multi-scan (*CrysAlis PRO*; Oxford Diffraction, 2006 ▶) *T*_min_ = 0.860, *T*_max_ = 1.0
6598 measured reflections
3031 independent reflections
2263 reflections with *I* > 2σ(*I*)
*R*_int_ = 0.014

#### Refinement

*R*[*F* ^2^ > 2σ(*F* ^2^)] = 0.036
*wR*(*F* ^2^) = 0.103
*S* = 1.13
3031 reflections
226 parameters
H-atom parameters constrained
Δρ_max_ = 0.31 e Å^−3^
Δρ_min_ = −0.34 e Å^−3^

### 

Data collection: *CrysAlis PRO* (Oxford Diffraction, 2006 ▶); cell refinement: *CrysAlis PRO*; data reduction: *CrysAlis PRO*; program(s) used to solve structure: *SHELXS97* (Sheldrick, 2008 ▶); program(s) used to refine structure: *SHELXL97* (Sheldrick, 2008 ▶); molecular graphics: *OLEX2* (Dolomanov *et al.*, 2009 ▶) and *Mercury* (Macrae *et al.*, 2006 ▶); software used to prepare material for publication: *OLEX2*.

## Supplementary Material

Crystal structure: contains datablocks I, global. DOI: 10.1107/S1600536811005915/gk2335sup1.cif

Structure factors: contains datablocks I. DOI: 10.1107/S1600536811005915/gk2335Isup2.hkl

Additional supplementary materials:  crystallographic information; 3D view; checkCIF report

## Figures and Tables

**Table 1:** Hydrogen-bond geometry (Å, °)

*D*—H⋯*A*	*D*—H	H⋯*A*	*D*⋯*A*	*D*—H⋯*A*
N1—H1*B*⋯S1^i^	0.86	2.69	3.4861 (16)	155
N1—H1*A*⋯O1^ii^	0.86	2.23	2.8654 (17)	130
N2—H2⋯O2	0.86	1.97	2.6708 (18)	138
N3—H3⋯O1^iii^	0.86	2.09	2.9062 (18)	157

Symmetry codes: (i) 
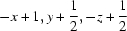
; (ii) 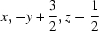; (iii) 
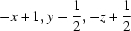
.

**Fig. 1.**
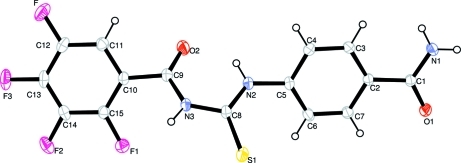
The molecular structure of the title compound, with displacement ellipsoids drawn at the 30% probability level.

**Fig. 2.**
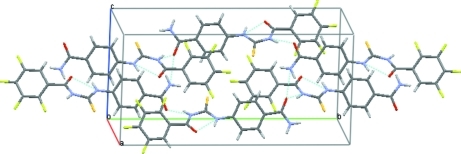
A packing diagram of the title compound, showing classical hydrogen bonds of N1—H1A···O1, N2—H2···O2 and N3—H3···O1 as green dashed lines.

## supplementary crystallographic information

### Comment

*N*-(4-Carbamoylphenylcarbamothioyl)-2,3,4,5-tetrafluorobenzamide derivatives are of great importance owing to their interesting biological properties (Zeng *et al.*, 2003; Saeed *et al.*, 2010). The title compound is one of the key intermediates in our synthetic route to antiviral drugs. We report here its crystal structure.

In the title compound, C_15_H_9_F_4_N_3_O_2_S, (Fig.1), the *cis,trans* geometry of the thiourea moiety is stabilized by intramolecular N2—H2···O2 and N3—H3···F1 hydrogen bonds. The central thiourea group makes dihedral angles of 47.79 (7) and 35.54 (8)° with the benzamide unit and the fluorobenzene ring, respectively. A combination of intermolecular π–π stacking interactions, N—H···O, N—H···F and N—H···S hydrogen bonds helps to stabilize the crystal structure (Table 1 and Fig.2).

### Experimental

A solution of 0.23 g (3 mmol) of ammonium thiocyanate in 7 ml of acetonitrile was added to a solution of 0.64 g (3 mmol) of 2,3,4,5-tetrafluorobenzoyl chloride in 2.5 ml of toluene. The mixture was heated for 5 min at 40°C and filtered from ammonium chloride, the filtrate was added to a solution of 0.32 g (3 mmol) of 4-aminobenzamide in 5 ml of acetonitrile, the mixture was stirred for 2 h at room temperature and evaporated, and the residue was washed with ethanol and recrystallized from ethanol. Yield 0.91 g (82%). Crystals suitable for X-ray analysis were obtained by slow evaporation from ethyl acetate solution.

### Refinement

All H atoms were positioned geometrically (C—H = 0.93 Å, N—H = 0.86 Å) and refined using a riding model approximation with *U*_iso_(H) = 1.2*U*_eq_(C,*N*).

### Crystal data

**Table:**

C_15_H_9_F_4_N_3_O_2_S	*F*(000) = 752
*M**_r_* = 371.31	*D*_x_ = 1.658 Mg m^−^^3^
Monoclinic, *P*2_1_/*c*	Mo *K*α radiation, λ = 0.7107 Å
Hall symbol: -P 2ybc	Cell parameters from 3669 reflections
*a* = 7.4246 (3) Å	θ = 3.3–29.2°
*b* = 20.3368 (7) Å	µ = 0.28 mm^−^^1^
*c* = 9.8954 (4) Å	*T* = 294 K
β = 95.554 (3)°	Block, colourless
*V* = 1487.12 (9) Å^3^	0.38 × 0.30 × 0.26 mm
*Z* = 4	

### Data collection

**Table:**

Oxford Diffraction Xcalibur E CCD diffractometer	3031 independent reflections
Radiation source: fine-focus sealed tube	2263 reflections with *I* > 2σ(*I*)
graphite	*R*_int_ = 0.014
Detector resolution: 16.0874 pixels mm^-1^	θ_max_ = 26.4°, θ_min_ = 3.4°
ω scans	*h* = −9→9
Absorption correction: multi-scan (*CrysAlis PRO*; Oxford Diffraction, 2006)	*k* = −25→21
*T*_min_ = 0.860, *T*_max_ = 1.0	*l* = −11→12
6598 measured reflections	

### Refinement

**Table:**

Refinement on *F*^2^	Primary atom site location: structure-invariant direct methods
Least-squares matrix: full	Secondary atom site location: difference Fourier map
*R*[*F*^2^ > 2σ(*F*^2^)] = 0.036	Hydrogen site location: inferred from neighbouring sites
*wR*(*F*^2^) = 0.103	H-atom parameters constrained
*S* = 1.13	*w* = 1/[σ^2^(*F*_o_^2^) + (0.056*P*)^2^] where *P* = (*F*_o_^2^ + 2*F*_c_^2^)/3
3031 reflections	(Δ/σ)_max_ < 0.001
226 parameters	Δρ_max_ = 0.31 e Å^−^^3^
0 restraints	Δρ_min_ = −0.34 e Å^−^^3^

### Special details

**Table:**

Geometry. All e.s.d.'s (except the e.s.d. in the dihedral angle between two l.s. planes) are estimated using the full covariance matrix. The cell e.s.d.'s are taken into account individually in the estimation of e.s.d.'s in distances, angles and torsion angles; correlations between e.s.d.'s in cell parameters are only used when they are defined by crystal symmetry. An approximate (isotropic) treatment of cell e.s.d.'s is used for estimating e.s.d.'s involving l.s. planes.
Refinement. Refinement of *F*^2^ against ALL reflections. The weighted *R*-factor *wR* and goodness of fit *S* are based on *F*^2^, conventional *R*-factors *R* are based on *F*, with *F* set to zero for negative *F*^2^. The threshold expression of *F*^2^ > σ(*F*^2^) is used only for calculating *R*-factors(gt) *etc*. and is not relevant to the choice of reflections for refinement. *R*-factors based on *F*^2^ are statistically about twice as large as those based on *F*, and *R*- factors based on ALL data will be even larger.

### Fractional atomic coordinates and isotropic or equivalent isotropic displacement parameters (Å^2^)

**Table:**

	*x*	*y*	*z*	*U*_iso_*/*U*_eq_	
S1	0.41600 (7)	0.40876 (2)	0.28889 (6)	0.05125 (18)	
F	0.98701 (18)	0.10215 (6)	−0.07009 (12)	0.0623 (4)	
F1	0.66951 (14)	0.21050 (5)	0.34645 (9)	0.0445 (3)	
F2	0.72451 (16)	0.08149 (5)	0.33952 (12)	0.0543 (3)	
F3	0.87569 (17)	0.02540 (5)	0.12899 (13)	0.0635 (4)	
O1	0.66762 (17)	0.73716 (6)	0.33958 (11)	0.0386 (3)	
O2	0.91223 (18)	0.33325 (6)	0.09914 (14)	0.0471 (3)	
N1	0.7405 (2)	0.75363 (7)	0.12865 (14)	0.0434 (4)	
H1B	0.7340	0.7956	0.1382	0.052*	
H1A	0.7683	0.7373	0.0532	0.052*	
N2	0.7175 (2)	0.43553 (7)	0.17165 (14)	0.0390 (4)	
H2	0.8046	0.4200	0.1300	0.047*	
N3	0.64026 (19)	0.32604 (7)	0.18763 (14)	0.0356 (3)	
H3	0.5580	0.2981	0.2036	0.043*	
C1	0.7076 (2)	0.71401 (8)	0.23066 (16)	0.0304 (4)	
C2	0.7164 (2)	0.64135 (8)	0.21097 (16)	0.0286 (4)	
C3	0.7548 (3)	0.61151 (9)	0.09123 (18)	0.0392 (4)	
H3A	0.7823	0.6372	0.0182	0.047*	
C4	0.7524 (3)	0.54374 (9)	0.07970 (18)	0.0425 (5)	
H4	0.7771	0.5241	−0.0013	0.051*	
C5	0.7133 (2)	0.50499 (8)	0.18833 (17)	0.0343 (4)	
C6	0.6795 (2)	0.53412 (8)	0.30877 (17)	0.0367 (4)	
H6	0.6566	0.5083	0.3828	0.044*	
C7	0.6796 (2)	0.60191 (8)	0.31958 (16)	0.0329 (4)	
H7	0.6546	0.6214	0.4007	0.039*	
C8	0.6006 (2)	0.39170 (8)	0.21371 (17)	0.0345 (4)	
C9	0.7920 (2)	0.30012 (9)	0.14025 (17)	0.0338 (4)	
C10	0.8043 (2)	0.22623 (8)	0.14010 (16)	0.0315 (4)	
C11	0.8887 (2)	0.19686 (9)	0.03581 (18)	0.0369 (4)	
H11	0.9315	0.2228	−0.0316	0.044*	
C12	0.9089 (2)	0.13022 (9)	0.03208 (19)	0.0410 (4)	
C13	0.8521 (3)	0.09051 (8)	0.1325 (2)	0.0414 (5)	
C14	0.7735 (2)	0.11852 (8)	0.23825 (18)	0.0369 (4)	
C15	0.7476 (2)	0.18583 (8)	0.24020 (16)	0.0326 (4)	

### Atomic displacement parameters (Å^2^)

**Table:**

	*U*^11^	*U*^22^	*U*^33^	*U*^12^	*U*^13^	*U*^23^
S1	0.0543 (3)	0.0289 (3)	0.0750 (4)	0.0028 (2)	0.0295 (3)	−0.0031 (2)
F	0.0717 (9)	0.0532 (7)	0.0638 (8)	0.0202 (6)	0.0162 (6)	−0.0171 (6)
F1	0.0550 (7)	0.0402 (6)	0.0394 (6)	0.0026 (5)	0.0093 (5)	0.0001 (5)
F2	0.0617 (8)	0.0366 (6)	0.0643 (7)	−0.0022 (5)	0.0052 (6)	0.0184 (5)
F3	0.0688 (8)	0.0237 (6)	0.0975 (10)	0.0083 (5)	0.0051 (7)	−0.0067 (6)
O1	0.0568 (8)	0.0293 (7)	0.0305 (6)	0.0036 (6)	0.0085 (6)	−0.0027 (5)
O2	0.0453 (8)	0.0312 (7)	0.0677 (9)	−0.0037 (6)	0.0199 (7)	0.0007 (6)
N1	0.0702 (11)	0.0250 (8)	0.0374 (8)	−0.0013 (7)	0.0174 (8)	0.0019 (7)
N2	0.0480 (9)	0.0228 (7)	0.0488 (9)	−0.0001 (7)	0.0181 (7)	−0.0001 (7)
N3	0.0392 (8)	0.0215 (7)	0.0481 (9)	0.0008 (6)	0.0145 (7)	0.0009 (6)
C1	0.0333 (9)	0.0274 (9)	0.0303 (9)	0.0002 (7)	0.0031 (7)	0.0003 (7)
C2	0.0310 (9)	0.0245 (9)	0.0303 (8)	0.0002 (7)	0.0028 (7)	0.0005 (7)
C3	0.0559 (12)	0.0280 (9)	0.0359 (10)	−0.0041 (8)	0.0166 (9)	0.0013 (8)
C4	0.0614 (12)	0.0293 (10)	0.0398 (10)	−0.0009 (9)	0.0206 (9)	−0.0057 (8)
C5	0.0409 (10)	0.0213 (9)	0.0416 (10)	−0.0004 (7)	0.0075 (8)	0.0002 (7)
C6	0.0509 (11)	0.0273 (9)	0.0319 (9)	−0.0022 (8)	0.0037 (8)	0.0056 (7)
C7	0.0432 (10)	0.0288 (9)	0.0269 (8)	−0.0004 (7)	0.0042 (7)	−0.0007 (7)
C8	0.0434 (10)	0.0233 (9)	0.0371 (9)	0.0023 (7)	0.0060 (8)	0.0010 (7)
C9	0.0368 (10)	0.0271 (9)	0.0377 (9)	0.0016 (7)	0.0045 (7)	0.0004 (7)
C10	0.0310 (9)	0.0252 (9)	0.0380 (9)	0.0018 (7)	0.0015 (7)	−0.0003 (7)
C11	0.0342 (9)	0.0341 (10)	0.0426 (10)	0.0040 (8)	0.0043 (8)	0.0013 (8)
C12	0.0387 (10)	0.0364 (10)	0.0475 (11)	0.0104 (8)	0.0022 (8)	−0.0113 (9)
C13	0.0389 (10)	0.0221 (9)	0.0612 (12)	0.0040 (8)	−0.0061 (9)	−0.0046 (9)
C14	0.0350 (10)	0.0275 (9)	0.0469 (10)	−0.0027 (7)	−0.0032 (8)	0.0060 (8)
C15	0.0296 (9)	0.0313 (9)	0.0366 (9)	0.0018 (7)	0.0022 (7)	−0.0011 (8)

### Geometric parameters (Å, °)

**Table:**

S1—C8	1.6583 (18)	C2—C7	1.389 (2)
F—C12	1.341 (2)	C3—H3A	0.9300
F1—C15	1.3458 (18)	C3—C4	1.383 (2)
F2—C14	1.332 (2)	C4—H4	0.9300
F3—C13	1.3365 (18)	C4—C5	1.386 (2)
O1—C1	1.2383 (18)	C5—C6	1.376 (2)
O2—C9	1.219 (2)	C6—H6	0.9300
N1—H1B	0.8600	C6—C7	1.383 (2)
N1—H1A	0.8600	C7—H7	0.9300
N1—C1	1.333 (2)	C9—C10	1.505 (2)
N2—H2	0.8600	C10—C11	1.393 (2)
N2—C5	1.423 (2)	C10—C15	1.384 (2)
N2—C8	1.338 (2)	C11—H11	0.9300
N3—H3	0.8600	C11—C12	1.364 (2)
N3—C8	1.397 (2)	C12—C13	1.378 (3)
N3—C9	1.367 (2)	C13—C14	1.370 (3)
C1—C2	1.493 (2)	C14—C15	1.383 (2)
C2—C3	1.385 (2)		
			
F—C12—C11	119.99 (18)	C4—C3—H3A	119.8
F—C12—C13	118.62 (16)	C4—C5—N2	117.77 (15)
F1—C15—C10	121.45 (14)	C5—N2—H2	116.5
F1—C15—C14	116.81 (15)	C5—C4—H4	119.8
F2—C14—C13	120.47 (15)	C5—C6—H6	120.1
F2—C14—C15	120.04 (16)	C5—C6—C7	119.84 (15)
F3—C13—C12	120.80 (18)	C6—C5—N2	122.40 (15)
F3—C13—C14	119.87 (18)	C6—C5—C4	119.77 (15)
O1—C1—N1	120.43 (15)	C6—C7—C2	120.96 (15)
O1—C1—C2	120.47 (14)	C6—C7—H7	119.5
O2—C9—N3	123.76 (16)	C7—C2—C1	117.15 (14)
O2—C9—C10	120.32 (15)	C7—C6—H6	120.1
N1—C1—C2	119.09 (14)	C8—N2—H2	116.5
H1B—N1—H1A	120.0	C8—N2—C5	127.09 (15)
N2—C8—S1	126.10 (13)	C8—N3—H3	115.6
N2—C8—N3	115.14 (15)	C9—N3—H3	115.6
N3—C8—S1	118.75 (12)	C9—N3—C8	128.85 (14)
N3—C9—C10	115.92 (14)	C10—C11—H11	119.9
C1—N1—H1B	120.0	C11—C10—C9	117.41 (15)
C1—N1—H1A	120.0	C11—C12—C13	121.38 (17)
C2—C3—H3A	119.8	C12—C11—C10	120.29 (17)
C2—C7—H7	119.5	C12—C11—H11	119.9
C3—C2—C1	124.10 (15)	C13—C14—C15	119.49 (16)
C3—C2—C7	118.74 (15)	C14—C13—C12	119.32 (15)
C3—C4—H4	119.8	C14—C15—C10	121.71 (15)
C3—C4—C5	120.31 (16)	C15—C10—C9	124.72 (15)
C4—C3—C2	120.34 (16)	C15—C10—C11	117.75 (15)
			
F—C12—C13—F3	0.6 (3)	C5—N2—C8—N3	−178.02 (15)
F—C12—C13—C14	179.40 (16)	C5—C6—C7—C2	−1.1 (3)
F2—C14—C15—F1	−1.0 (2)	C7—C2—C3—C4	1.4 (3)
F2—C14—C15—C10	177.00 (15)	C8—N2—C5—C4	−138.63 (19)
F3—C13—C14—F2	1.7 (3)	C8—N2—C5—C6	44.1 (3)
F3—C13—C14—C15	−179.20 (15)	C8—N3—C9—O2	−8.1 (3)
O1—C1—C2—C3	178.02 (16)	C8—N3—C9—C10	172.27 (16)
O1—C1—C2—C7	−0.5 (2)	C9—N3—C8—S1	−172.62 (14)
O2—C9—C10—C11	−33.5 (2)	C9—N3—C8—N2	8.7 (3)
O2—C9—C10—C15	142.48 (18)	C9—C10—C11—C12	178.10 (15)
N1—C1—C2—C3	−0.9 (3)	C9—C10—C15—F1	2.2 (2)
N1—C1—C2—C7	−179.44 (15)	C9—C10—C15—C14	−175.74 (15)
N2—C5—C6—C7	179.11 (16)	C10—C11—C12—F	178.65 (16)
N3—C9—C10—C11	146.16 (16)	C10—C11—C12—C13	−2.0 (3)
N3—C9—C10—C15	−37.9 (2)	C11—C10—C15—F1	178.11 (14)
C1—C2—C3—C4	−177.14 (16)	C11—C10—C15—C14	0.2 (2)
C1—C2—C7—C6	178.11 (16)	C11—C12—C13—F3	−178.74 (16)
C2—C3—C4—C5	−0.6 (3)	C11—C12—C13—C14	0.1 (3)
C3—C2—C7—C6	−0.5 (3)	C12—C13—C14—F2	−177.14 (15)
C3—C4—C5—N2	−178.38 (17)	C12—C13—C14—C15	2.0 (3)
C3—C4—C5—C6	−1.0 (3)	C13—C14—C15—F1	179.87 (15)
C4—C5—C6—C7	1.9 (3)	C13—C14—C15—C10	−2.1 (3)
C5—N2—C8—S1	3.4 (3)	C15—C10—C11—C12	1.9 (2)

### Hydrogen-bond geometry (Å, °)

**Table:**

*D*—H···*A*	*D*—H	H···*A*	*D*···*A*	*D*—H···*A*
N1—H1B···S1^i^	0.86	2.69	3.4861 (16)	155
N1—H1A···O1^ii^	0.86	2.23	2.8654 (17)	130
N2—H2···O2	0.86	1.97	2.6708 (18)	138
N3—H3···F1	0.86	2.37	2.8234 (17)	113
N3—H3···O1^iii^	0.86	2.09	2.9062 (18)	157

Symmetry codes: (i) −*x*+1, *y*+1/2, −*z*+1/2; (ii) *x*, −*y*+3/2, *z*−1/2; (iii) −*x*+1, *y*−1/2, −*z*+1/2.
